# Immunoregulatory Mechanisms Underlying Prevention of Colitis-Associated Colorectal Cancer by Probiotic Bacteria

**DOI:** 10.1371/journal.pone.0034676

**Published:** 2012-04-12

**Authors:** Josep Bassaganya-Riera, Monica Viladomiu, Mireia Pedragosa, Claudio De Simone, Raquel Hontecillas

**Affiliations:** 1 Nutritional Immunology and Molecular Medicine Laboratory, Center for Modeling Immunity to Enteric Pathogens, Virginia Bioinformatics Institute, Virginia Tech, Blacksburg, Virginia, United States of America; 2 Experimental Medicine, L'Aquila University, L'Aquila, Italy; University of Aberdeen, United Kingdom

## Abstract

**Background:**

Inflammatory bowel disease (IBD) increases the risk of colorectal cancer. Probiotic bacteria produce immunoregulatory metabolites *in vitro* such as conjugated linoleic acid (CLA), a polyunsaturated fatty acid with potent anti-carcinogenic effects. This study aimed to investigate the cellular and molecular mechanisms underlying the efficacy of probiotic bacteria in mouse models of cancer.

**Methodology/Principal Findings:**

The immune modulatory mechanisms of VSL#3 probiotic bacteria and CLA were investigated in mouse models of inflammation-driven colorectal cancer. Colonic specimens were collected for histopathology, gene expression and flow cytometry analyses. Immune cell subsets in the mesenteric lymph nodes (MLN), spleen and colonic lamina propria lymphocytes (LPL) were phenotypically and functionally characterized. Mice treated with CLA or VSL#3 recovered faster from the acute inflammatory phase of disease and had lower disease severity in the chronic, tumor-bearing phase of disease. Adenoma and adenocarcinoma formation was also diminished by both treatments. VSL#3 increased the mRNA expression of TNF-α, angiostatin and PPAR γ whereas CLA decreased COX-2 levels. Moreover, VSL#3-treated mice had increased IL-17 expression in MLN CD4+ T cells and accumulation of Treg LPL and memory CD4+ T cells.

**Conclusions/Significance:**

Both CLA and VSL#3 suppressed colon carcinogenesis, although VSL#3 showed greater anti-carcinogenic and anti-inflammatory activities than CLA. Mechanistically, CLA modulated expression of COX-2 levels in the colonic mucosa, whereas VSL#3 targeted regulatory mucosal CD4+ T cell responses.

## Introduction

Colorectal cancer (CRC) is the third most commonly diagnosed cancer in the United States [1]. An estimated 50,000 deaths are attributed to CRC around the world each year [2]. Together with the hereditary syndromes of familial adenomatous polyposis and hereditary nonpolyposis, inflammatory bowel disease (IBD) is among the top three high-risk conditions for CRC [3]. Among ulcerative colitis (UC) patients, one of the two main manifestations of IBD, the relative risk of developing CRC correlates with the extent and duration of disease [3], [4]. In patients with IBD, this risk increases by 0.5–1.0% yearly after 8–10 years [5].

The human gut microflora, which contains about 100 trillion microbial organisms, plays a critical role in maintaining host health, both in the gastrointestinal tract and systemically through the absorption of metabolites (e.g. vitamins and short chain fatty acids) [6]. Recent studies have demonstrated that specific strains of bacteria are implicated in the regulation of the intestinal homeostasis, delivering regulatory signals to the epithelium, the mucosal immune system and to the neuromuscular activity of the gut [7], [8]. Moreover, some commensal and pathogenic organisms of the human enteric microbiome are essential in the pathogenesis of IBD and CRC. Therefore, manipulating the gut bacterial composition and local metabolite production by using probiotic bacteria has been explored as a promising avenue for therapeutic intervention against CRC. Probiotics are live microbial feed supplements which beneficially impact on host health. They rely on introducing particular exogenous strains into the gut microflora [8], [9]. These strains are often chosen for specific beneficial activities, like the production of lipids such as conjugated linoleic acid (CLA) and conjugated linoleic acid isomers (CLNA) [10], [11]. These lipids vary in chemical structure from short fatty acids such as propionate, acetate and butyrate to polyunsaturated fatty acids (PUFA) and are involved in regulating apoptosis and the immune response [10], [12]–[14].

A screening of 36 different Bifidobacterium strains for their ability to produce CLA from free linoleic acid and CLNA from alpha-linolenic acid (LNA) reveals that six strains (four *Bifidobacterium breve* strains, a *B. bifidum* strain and a *B. pseudolongum* strain) are able to produce different CLA and CLNA isomers *in vitro* [15]. Others have shown that probiotic bacteria can synthesize CLA [16]–[18]. CLA-producing bacterial strains can be found in a commercially available probiotic formulation known as VSL#3. This product is composed of four strains of lactobacilli (*Lactobacillus casei*, *L. plantarum*, *L. bulgaricus*, and *L. acidophilus*), three strains of bifidobacteria (*Bifidobacterium longum*, *B. breve*, *and B. infantis*) and *Streptococcus thermophilus*. Recently, we have demonstrated that VSL#3 probiotic bacteria produce CLA locally that targets macrophage peroxisome proliferator-activated receptor (PPAR) γ to suppress colitis [19]. In addition, VSL#3 may play a key role in maintaining the intestinal microbial balance by synthesizing antibacterial substances like lantibiotics [20], and other bacteriocin-like compounds [21]. Moreover, VSL#3 treatment is associated with a down-regulation of LPS-activated IL-8 production and suppressed secretion of TNF-α and IFN-γ [22]. These immunoregulatory actions are consistent with activation of PPAR γ by the gut microflora following VSL#3 probiotic treatment. The objective of this study is to investigate the ability of VSL#3 bacteria to modulate mucosal immune responses and thereby ameliorate colonic carcinogenesis.

## Materials and Methods

### Animal procedures and experimental diets

C57BL/6 wild-type mice (n = 60) were used for DSS-induced CRC study. In a follow up study, we also used IL-10-deficient (IL-10−/−) and wild-type mice in a 129/SvEv background (n = 60). For each experiment, mice were fed purified AIN-93G rodent diets (Table S1) with or without 1% CLA for 24 days prior the induction of inflammation-related cancer, in which all the requirements where met or exceeded. The CLA supplement administered contained a 50∶50 mixture of the *cis*-9, *trans*-11 CLA and *trans*-10, *cis*-12 isomers (Clarinol, Loders Croklaan BV).

### Ethics Statement

All experimental procedures were approved by the Virginia Tech Institutional Animal Care and Use Committee (IACUC) and met or exceeded requirements of the Public Health Service/National Institutes of Health and the Animal Welfare Act.

### Oral treatment with probiotic bacteria

For each experiment, approximately half of the mice received 0.5 mL of the VSL#3 probiotic solution daily by orogastric gavage using a ball tip gavage needle. The probiotic solution was freshly prepared daily in phosphate buffered saline (PBS) at pH 7.1 in sterile conditions to a final concentration of 0,0072 g VSL#3/mL, corresponding to 1.2 billion bacteria per mouse/day, a dose that has shown efficacy in colonizing the colon and terminal ileum [23], [24]. Further, this dose is biologically relevant since it is based on a daily intake of about 3,600 billion bacteria for an adult human weighing 70 kg. VSL#3 is a commercial probiotic mixture composed of four strains of lactobacilli (*Lactobacillus casei*, *L. plantarum*, *L. bulgaricus*, and *L. acidophilus*), three strains of bifidobacteria (*Bifidobacterium longum, B. breve, and B. infantis*) and *Streptococcus thermophilus*.

### Colorectal cancer induction

A previously established protocol was followed to chemically induce inflammation-driven CRC [25], [26]. Wild type mice were challenged with azoxymethane (10 mg/kg) on week 6 of the study, followed by a 2.0% dextran sodium sulfate (DSS) treatment in the drinking water for 7 days beginning on week 7. IL-10-deficient 129/SvEv mice were challenged with a single dose of 5×10^7^ cfu *Helicobacter typhlonius* by oral gavage to accelerate colon carcinogenesis as previously shown [27]. Disease activity indices and rectal bleeding scores were calculated using a modification of a previously published compounded score [28]. Mice were euthanized at the moment of tumor formation, corresponding to day 68 (C57BL/6 strain) or day 18 after infection with *H. typhlonius* (129/SvEv strain).

### Histopathology

Colonic sections were fixed in 10% buffered neutral formalin, later embedded in paraffin, and then sectioned (6 µm) and stained with H&E for histological examination. Tissue slides were examined in an Olympus microscope (Olympus America Inc., Dulles, VA). Colons were scored for leukocyte infiltration, epithelial erosion, mucosal thickness, adenocarcinomas and adenomas.

### RNA isolation and real-time polymerase chain reaction of cytokines

Total RNA from colon was isolated using the Qiagen RNA isolation kit (Qiagen) according to the manufacturer's instructions, and then was used to generate the cDNA template using the iScript cDNA synthesis kit (Bio-Rad, Hercules, CA) and real-time RT-PCR was performed as previously described [12].

### Immunophenotyping

Spleen and mesenteric lymph nodes (MLN) were excised and single-cell suspensions of tissues were resuspended in PBS and enumerated with the Coulter Counter (Beckman Coulter, Fullerton, CA). Colon samples were processed for lamina propria lymphocyte (LPL) isolation. Specifically, cells (6×10^5^ cells/well) were seeded into 96 well-plates, centrifuged at 4°C at 3000 rpm for 3 minutes, and washed with PBS containing 5% serum and 0,09% sodium azide (FACS buffer). Cells were then incubated for macrophage assessment with fluorochrome-conjugated primary antibodies to T cell and macrophage markers.

### Statistical analysis

To determine the statistical significance of the model, analysis of variance (ANOVA) was performed using the general linear model procedure of Statistical Analysis Software (SAS), and probability value (*P*)<0.05 was considered to be significant. When the model was significant, ANOVA was followed by Fisher's Protected Least Significant Difference multiple comparison method.

## Results

### VSL#3 and CLA ameliorate disease activity in mice with CRC

**Figure 1:**
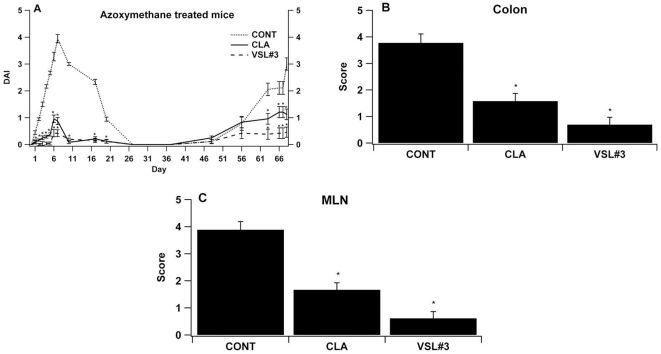
Effect of VSL#3 and dietary conjugated linoleic acid (CLA) supplementation on experimental azoxymethane-induced colorectal cancer. C57BL/6J mice (n = 60) were treated with the VSL#3 probiotic (n = 20), CLA-supplemented (1 g/100 g) (n = 20) or control diets (n = 20) for 32 days and challenged i.p. with azoxymethane (10 mg/kg) followed by 2% dextran sodium sulfate (DSS) in the drinking water for 7 days to induce colitis-associated colorectal cancer (CRC). The disease activity index, a composite score reflecting clinical signs of the disease (i.e. perianal soiling, rectal bleeding, diarrhea, and piloerection) was assessed daily for mice undergoing the DSS challenge (A). Mice were euthanized on day 68. Colon and mesenteric lymph nodes (MLN) (B&C) were macroscopically scored for inflammation. Data are represented as mean ± standard error. Points with an asterisk are significantly different when compared to the control group (*P*<0.05).

**Figure 2:**
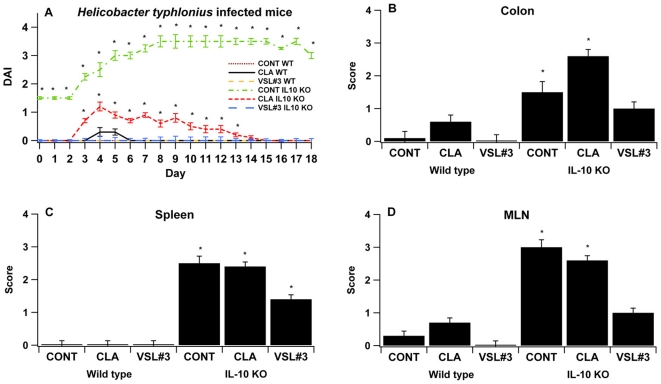
Effect of VSL#3 and dietary conjugated linoleic acid (CLA) supplementation on experimental *Helicobacter typhlonius*-induced colorectal cancer. Bacterial-free 129/SvEv and IL-10 gene deficient (IL-10−/−) 129/SvEv mice in a 129/SvEv background (n = 60) were treated with the VSL#3 probiotic (n = 20), CLA-supplemented (1 g/100 g) (n = 20) or control diets (n = 20) for 32 days and then were infected with *H. typhlonius* in order to develop experimental colorectal cancer associated with colitis. The disease activity index, a composite score reflecting clinical signs of the disease, was assessed daily for mice undergoing the DSS challenge (A). Colon, spleen and mesenteric lymph nodes (MLN) (B–D) were macroscopically scored for inflammation. Data are represented as mean ± standard error. Points with an asterisk are significantly different when compared to the control group (*P*<0.05).

In both models, VSL#3 and CLA treatments decreased disease activity scores associated with colitis-associated CRC in comparison to the untreated control group. In the azoxymethane-induced CRC model, mice treated with either CLA or VSL#3 recovered faster from the acute inflammatory phase of disease (days 1–26) and had lower disease severity in the chronic, tumor-bearing phase of disease (days 63–68). Overall, VSL#3 probiotic bacteria treatment was more effective than CLA in decreasing inflammation and reducing disease activity in both models (Figure 1A and Figure 2A). In line with these clinical findings, VSL#3 and CLA significantly ameliorated gross pathology in colon, spleen and MLN in comparison to untreated control mice in the azoxymethane induced CRC (Figure 1B–C). In the IL10−/− model, the probiotic mixture VSL#3 but not CLA decreased inflammation-related lesions in MLN, colon and spleen of *H. typhlonius* infected mice in comparison to the control group (Figure 2B–D).

### VSL#3 and CLA prevent colonic histological lesions in mice with CRC

**Figure 3:**
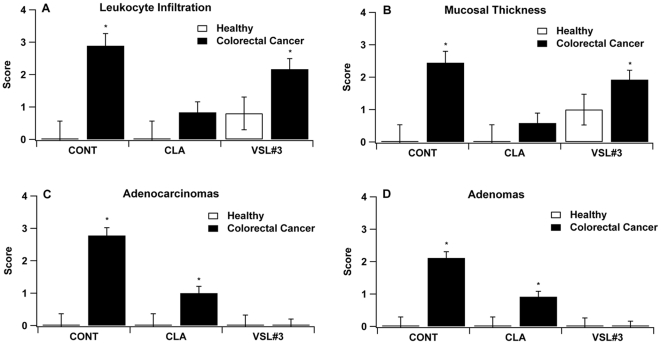
Effect of the CLA and VSL#3 treatment on colon histopathology on experimental azoxymethane-induced colorectal cancer. C57BL/6J mice (n = 60) were treated with the VSL#3 probiotic (n = 20), CLA-supplemented (1 g/100 g) (n = 20) or control diets (n = 20) for 32 days and challenged i.p. with azoxymethane (10 mg/kg) followed by 2% dextran sodium sulfate (DSS) in the drinking water for 7 days to induce colitis-associated colorectal cancer (CRC). Mice were euthanized on day 68. All specimens underwent blinded histological examination and were scored 1–4 on leukocyte infiltration (A), and mucosal wall thickening (B), adenocarcinomas (C) and adenomas (D). Data are represented as mean ± standard error. Points with an asterisk are significantly different when compared to the control group (*P*<0.05).

**Figure 4:**
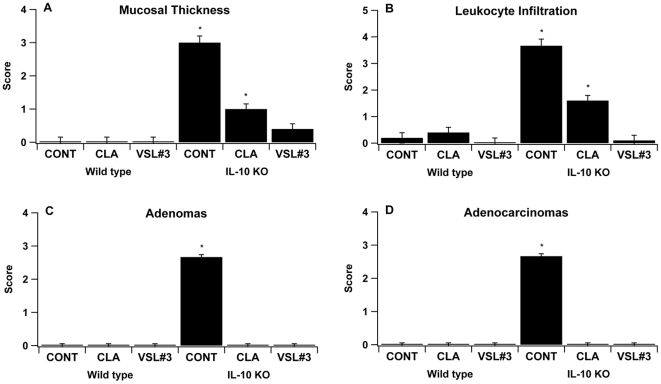
Effect of the CLA and VSL#3 treatment on colon histopathology on experimental *Helicobacter typhlonius*-induced colorectal cancer. Bacterial-free 129/SvEv and IL-10 gene deficient (IL-10−/−) 129/SvEv mice in a 129/SvEv background (n = 60) were treated with the VSL#3 probiotic (n = 20), CLA-supplemented (1 g/100 g) (n = 20) or control diets (n = 20) for 32 days and then were infected with *H. typhlonius* in order to develop experimental colorectal cancer associated with colitis. After the necropsy, all specimens underwent blinded histological examination and were scored 1–4 on mucosal wall thickening (A), leukocyte infiltration (B), adenomas (C) and adenocarcinomas (D). Data are represented as mean ± standard error. Points with an asterisk are significantly different when compared to the control group (*P*<0.05).

Regarding the azoxymethane/DSS colitis-associated CRC model, CLA treatment, but not VSL#3 administration, decreased leukocyte infiltration and colonic mucosal thickness (Figure 3 A&B). Interestingly, VSL#3 was more effective than CLA in reducing leukocyte infiltration in mucosal thickness in IL-10−/− mice infected with *H. typhlonius* (Figure 4 A&B). Additionally, both VSL#3 and CLA treatments diminished adenoma and adenocarcinoma formation when compared to the control. These results were consistent for both CRC models (Figure 3 C&D and Figure 4 C&D).

### VSL#3 and CLA modulate expression of colonic inflammatory and carcinogenesis markers

**Figure 5:**
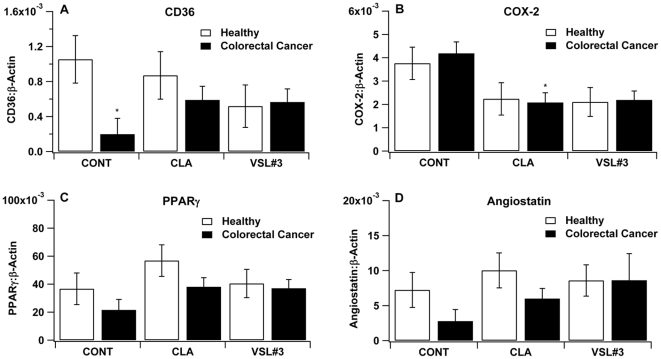
VSL#3 and conjugated linoleic acid (CLA) modulate colonic gene expression. C57BL/6J mice (n = 60) were treated with the VSL#3 probiotic (n = 20), CLA-supplemented (1 g/100 g) (n = 20) or control diets (n = 20) for 32 days and challenged i.p. with azoxymethane (10 mg/kg) followed by 2% dextran sodium sulfate (DSS) in the drinking water for 7 days to induce colitis-associated colorectal cancer (CRC). Mice were euthanized on day 68. Expression of CD36 (A), cyclooxygenase 2 (COX2) (B), peroxisome proliferator-activated receptor γ (PPAR γ) (C) and angiostatin (D) were assessed by real-time quantitative PCR. Data are represented as mean ± standard error. Points with an asterisk are significantly different when compared to the control group (*P*<0.05).

Mice challenged with azoxymethane and DSS were euthanized in the tumor-bearing phase of the disease. Those mice treated with either CLA or VSL#3 showed an increased mRNA expression of CD36 and PPAR γ. in the colon (Figure 5 A&C). In addition, oral CLA administration significantly down-regulated colonic COX-2 expression (Figure 5B) whereas VSL#3 treatment upregulated angiostatin mRNA levels in the distal colon (Figure 5D), a proteolytic fragment of plasminogen with anti-angiogenic effects in mice with CRC. Finally, an upregulation of colonic TNF-α was found in CLA and VSL#3-treated mice when compared to the untreated control group (data not shown).

### VSL#3 and CLA modulate phenotype and cytokine production of macrophages and CD4+ T cell subsets in the MLN and colonic LP

**Figure 6:**
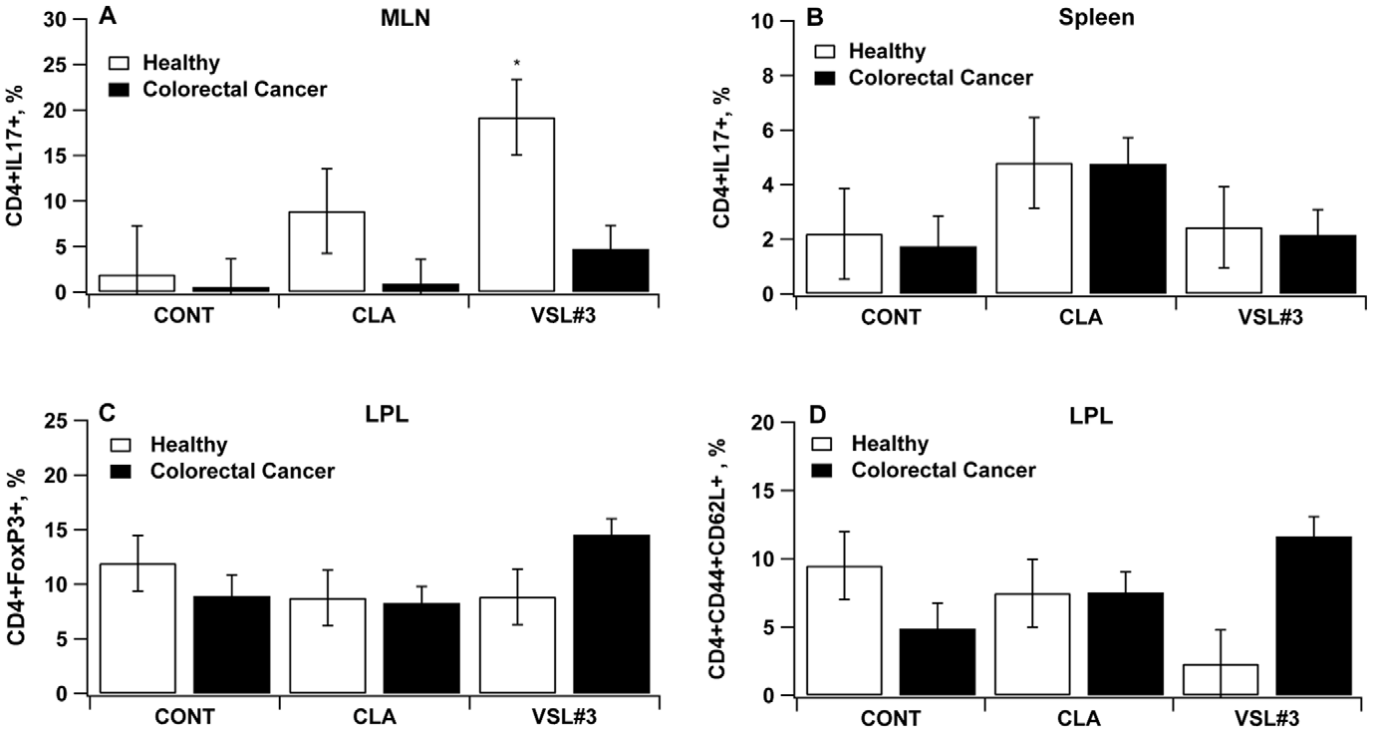
VSL#3 and conjugated linoleic acid (CLA) modulate immune cell subsets in mesenteric lymph nodes (MLN), colonic lamina propria lymphocytes (LPL) and spleen. C57BL/6J mice (n = 60) were treated with the VSL#3 probiotic (n = 20), CLA-supplemented (1 g/100 g) (n = 20) or control diets (n = 20) for 32 days and challenged i.p. with azoxymethane (10 mg/kg) followed by 2% dextran sodium sulfate (DSS) in the drinking water for 7 days to induce colitis-associated colorectal cancer (CRC). Mice were euthanized on day 68. MLN (A–C), spleen (D) and LPL (E–F) from wild type mice were immunophenotyped to identify immune cells subsets by flow cytometry. Data are represented as mean ± standard error. Points with an asterisk are significantly different when compared to the control group (*P*<0.05).

Those healthy mice that received VSL#3 had greater percentages of IL-17-expressing CD4+ T cells in MLN when compared to the untreated control group (Figure 6A). Similarly, CLA treatment increased the population of IL-17-expressing CD4+ T cells in the spleen of both healthy and CRC mice (Figure 6B). Greater percentages of CD4^+^FoxP3^+^ and CD4^+^CD44^+^CD62L^+^ LP T cells, corresponding to mucosal regulatory T cells (Treg) and CD4^+^ memory T cells respectively, were found in VSL#3-treated mice (Figure 6C&D).

## Discussion

Chronic intestinal inflammation occurring in IBD induces persistent damage and enhanced mucosal permeability along the gastrointestinal tract, playing an important role in the development of colorectal cancer (CRC) [29]. Manipulation of colonic bacteria with probiotics has been shown to be effective in the regulation of gut homeostasis in part through production of bacterial metabolites [30]. VSL#3 probiotic bacteria can produce CLA locally in the gut, a compound that down-modulates inflammatory responses by activating PPAR γ [31]. In addition, CLA prevents or ameliorates experimental IBD in mice and pigs [12], [13], [32]. Moreover, PPAR γ agonists have shown clinical efficacy against human UC [33], [34]. Recently, we demonstrated that the probiotic mixture VSL#3 suppresses intestinal inflammation by producing CLA locally in the colon and activating PPAR γ in macrophages [19]. This study aimed to investigate the mechanisms of CLA and VSL#3 in mouse models of colitis-associated CRC.

The clinical response in the AOM/DSS model had a bimodal distribution in which the first peak corresponded to acute inflammation due to the DSS challenge followed by a period of clinical recovery characterized by lower chronic inflammation. The second peak of disease appeared on day 48 corresponding to the tumor formation. This pattern is consistent with results of previous studies from our group that characterized the anti-carcinogenic properties of CLA [25]. CLA and VSL#3 treated mice also showed lower macroscopic inflammation-related lesions and significantly improved colonic histopathology in both experimental models. Nevertheless, while VSL#3 was the compound that prevented tumor formation most effectively, it did not prevent leukocyte infiltration in comparison to the other groups in the azoxymethane-induced colorectal cancer model, suggesting a possible role of infiltrating leukocytes in the anti-carcinogenic actions of VSL#3. In a follow up study, using a different model of colitis-associated colonic carcinogenesis, IL-10−/− mice in a 129/SvEv background were infected with *H. typhlonius*, thus accelerating the colonic inflammation that the IL10−/− develop spontaneously and promoting colonic carcinogenesis [27]. In this model, both VSL#3 and CLA ameliorated disease severity but only the probiotic mixture was effective in reducing inflammation-related lesions in MLN, spleen and colon. This consistency of results in both models demonstrates that the efficacy of VSL#3 in restoring mucosal homeostasis and attenuating colitis-associated CRC is not model-dependent.

Our gene expression analyses showed an upregulation of colonic TNF-α in CLA and VSL#3-treated mice when compared to the untreated control group, which may be indicative of immunostimulation and enhanced epithelial healing ability. Most notably, TNF-α exerts potent antitumoral effects by stimulating immune responses, including upregulation of human leukocyte antigen antigens in tumor cell surfaces [35], enhanced cytotoxicity [36] stimulation of cytotoxic T cells and natural killer (NK) cells [37]–[40] as well as epithelial healing [41]. In addition, CLA-treated mice showed a significantly reduced expression of cyclooxygenase-2 (COX-2), an enzyme involved in arachidonic acid cascade and prostaglandin-mediated inflammation. Previous studies have suggested the suppressive effects of CLA on colon carcinogenesis due to changes in this cascade and in the activation of PPAR γ, both mechanisms involving the inhibition of COX-2 expression and inducing apoptosis [42], [43]. The levels of angiostatin, a proteolytic fragment of plasminogen and an endogenous inhibitor of angiogenesis [44], were greater in the VSL#3 group when compared to control fed mice, suggesting that the anti-carcinogenic actions of VSL#3 may be mediated, in part, by suppressed angiogenesis. As shown in previous studies [45], [46], this inhibitory behavior of angiostatin on angiogenesis could be indirectly related with anti-carcinogenic efficacy. Our findings extend the findings of a recent report demonstrating that probiotic VSL#3 can attenuate chronic inflammation in rats, delaying the transition from inflammation to dysplasia and cancer [47].

The ability of VSL#3 and CLA to modulate immune responses and prevent CRC was also assessed by examining the distribution of immune cell subsets at the colonic mucosa and systemically. VSL#3 treatment enhanced the percentages of IL-17-expressing CD4+ T cells in the mucosal inductive site (i.e., MLN) and Foxp3-expressing CD4+ T cells in the effector site in mice with CRC, suggesting a possible role in modulating the plasticity between Th17 and Treg in the MLN and colonic LP. Results from a recent study identified segmented filamentous bacteria in the gut commensal microbiota as inducers of the Th17 polarization in the gut mucosa [48]. It is tempting to speculate that changes in the composition of the colon microbiome triggered by probiotic bacteria may exert similar effects. In conclusion, our data demonstrate the ability of CLA and VSL#3 to ameliorate inflammation-induced colorectal cancer through a mechanism involving modulation of mucosal CD4+ T cell polarization and modulation of gene expression.

## Supporting Information

Table S1 **Composition of experimental diets.** ^1^ Per kg diet: 3 g nicotinic acid, 1.6 g calcium pantotenate, 0.7 g pyridoxine HCl, 0.6 g thiamin HCl, 0.6 g riboflavin, 0.2 g folic acid, 0.02 g D-biotin, 2.5 g vitamin B-12 (0.1% in mannitol), 15 g d,l-α tocopheryl acetate (500 IU/g), 0.8 g vitamin A palmitate (500,000 IU/g), 0.2 g cholecalciferol (500,000 IU/g), 0.075 g vitamin K (phylloquinone), and 974.705 g sucrose. ^2^ Per kg diet: 357 g calcium carbonate, 196 g potassium phosphate monobasic, 70.78 g potassium citrate, 74 g sodium chloride, 46.6 g potassium sulfate, 24.3 g magnesium oxide, 6.06 g ferric citrate, 1.65 g zinc carbonate, 0.63 g manganous carbonate, 0.31 g cupric carbonate, 0.01 g potassium iodate, 0.01025 g sodium selenate, 0.00795 g ammonium paramolybdate, 1.45 g sodium meta-silicate, 0.275 g chromium potassium sulfate, 0.0174 g lithium chloride, 0.0815 g boric acid, 0.0635 g sodium fluoride, 0.0318 g nickel carbonate, hydroxide, tetrahydrate, 0.0066 g ammonium vanadate, and 220.716 g sucrose. ^3^Antioxidant.
(DOCX)
