# Correction: Immunoregulatory Mechanisms Underlying Prevention of Colitis-Associated Colorectal Cancer by Probiotic Bacteria

**DOI:** 10.1371/journal.pone.0275512

**Published:** 2022-09-27

**Authors:** The PLOS ONE Editors

The Competing Interests statement for this article [1] is incomplete. Based on information provided in editorial follow up, an updated Competing Interests statement is as follows: At the time of publication of this study, the probiotic formulation sold as VSL#3 was produced by Dupont/Danisco in the US. Claudio De Simone (CDS) is the inventor of this probiotic formulation (also known as the De Simone Formulation). CDS is named as an inventor and assignee on patent “Dietary and pharmaceutical compositions containing lyophilized lactic bacteria, their preparation and use” (U.S. Patent No. 5,716,615) and the sole owner of the know-how used to formulate the product. At the time of publication of the article, CDS owned one share of stock in, and served as Chief Executive Officer and Director for, VSL Pharmaceuticals, Inc., which sold the De Simone Formulation until 2016. At the time of publication of this study, co-author Josep Bassaganya-Riera was a *PLOS ONE* Editorial Board member. This does not alter the authors’ adherence to all the *PLOS ONE* policies on sharing data and materials.

The VSL#3 used in this study was obtained from VSL Pharmaceuticals and produced by Danisco/Dupont in the US.

There is an error in reference 10. The correct reference is: Hofmanova J, Ciganek M, Slavik J, Kozubik A, Stixova L, et al. (2011) Lipid alterations in human colon epithelial cells induced to differentiation and/or apoptosis by butyrate and polyunsaturated fatty acids. J Nutr Biochem. Volume 23, Issue 6, June 2012, Pages 539–548

There is an error in reference 15. The correct reference is: Gorissen L, Raes K, Weckx S, Dannenberger D, Leroy F, et al. (2010) Production of conjugated linoleic acid and conjugated linolenic acid isomers by Bifidobacterium species. Applied Microbiology and Biotechnology, August 2010, Volume 87, Issue 6, pp 2257–2266

There is an error in reference 19. The correct reference is: Bassaganya-Riera J, Viladomiu M, Pedragosa M, De Simone C, Carbo A, Shaykhutdinov R, et al. (2012) Probiotic Bacteria Produce Conjugated Linoleic Acid Locally in the Gut That Targets Macrophage PPAR γ to Suppress Colitis. PLoS ONE 7(2): e31238. https://doi.org/10.1371/journal.pone.0031238

There is an error in reference 44. The correct reference is: Shantha Kumara HM, Tohme ST, Yan X, Nasar A, Senagore AJ, et al. (2010) Plasma levels of angiostatin and endostatin remain unchanged for the first 3 weeks after colorectal cancer surgery. Surgical Endoscopy June 2011, Volume 25, Issue 6, pp 1939–1944

There is an error in reference 47. The correct reference is: Appleyard CB, Cruz ML, Isidro AA, Arthur JC, Jobin C, et al. (2011) Pretreatment with the Probiotic Vsl#3 Delays Transition from Inflammation to Dysplasia in a Rat Model of Colitis-Associated Cancer. Am J Physiol Gastrointest Liver Physiol. 301: G1004–G1013. doi:10.1152/ajpgi.00167.2011
