# Supplementary material for: Immunoregulatory Mechanisms Underlying Prevention of Colitis-Associated Colorectal Cancer by Probiotic Bacteria
Source: PLoS One. 2012 Apr 12;7(4):e34676. doi: 10.1371/journal.pone.0034676 (PMC3325233; doi:10.1371/journal.pone.0034676)
Supplement: Table S1 — Composition of experimental diets. 1 Per kg diet: 3 g nicotinic acid, 1.6 g calcium pantotenate, 0.7 g pyridoxine HCl, 0.6 g thiamin HCl, 0.6 g riboflavin, 0.2 g folic acid, 0.02 g D-biotin, 2.5 g vitamin B-12 (0.1% in mannitol), 15 g d,l-α tocopheryl acetate (500 IU/g), 0.8 g vitamin A palmitate (500,000 IU/g), 0.2 g cholecalciferol (500,000 IU/g), 0.075 g vitamin K (phylloquinone), and 974.705 g sucrose. 2 Per kg diet: 357 g calcium carbonate, 196 g potassium phosphate monobasic, 70.78 g potassium citrate, 74 g sodium chloride, 46.6 g potassium sulfate, 24.3 g magnesium oxide, 6.06 g ferric citrate, 1.65 g zinc carbonate, 0.63 g manganous carbonate, 0.31 g cupric carbonate, 0.01 g potassium iodate, 0.01025 g sodium selenate, 0.00795 g ammonium paramolybdate, 1.45 g sodium meta-silicate, 0.275 g chromium potassium sulfate, 0.0174 g lithium chloride, 0.0815 g boric acid, 0.0635 g sodium fluoride, 0.0318 g nickel carbonate, hydroxide, tetrahydrate, 0.0066 g ammonium vanadate, and 220.716 g sucrose. 3Antioxidant. (DOCX) [file pone.0034676.s001.docx]

| ^[1](#_ENREF_38)^**Ingredient** | **Control diet (g/Kg)** | **CLA diet (g/Kg)** |
| --- | --- | --- |
| **Casein** | 200 | 200 |
| **L-Cystein** | 3 | 3 |
| **Corn starch** | 397.5 | 397.5 |
| **Maltodextrin** | 132 | 132 |
| **Sucrose** | 100 | 100 |
| **Cellulose** | 50 | 50 |
| **Mineral mix (AIN-93)^1^** | 35 | 35 |
| **Vitamin mix (AIN-93)^2^** | 10 | 10 |
| **Choline bitartrate** | 2.5 | 2.5 |
| **Tert-butylhydroquinone^3^** | 0.014 | 0.014 |
| **Soybean oil** | 70 | 60 |
| **cis9, trans11/trans10, cis12 CLA (50:50)** | 0 | 10 |
